# Comparing 90-Day Postoperative Mortality After Neoadjuvant Proton-Based Versus Photon-Based Chemoradiotherapy for Esophageal Cancer

**DOI:** 10.1016/j.ijpt.2024.100012

**Published:** 2024-04-24

**Authors:** Pim J.J. Damen, Peter S.N. van Rossum, Yiqing Chen, Chike O. Abana, Zhongxing Liao, Brian P. Hobbs, Radhe Mohan, Mariela Blum-Murphy, Wayne L. Hofstetter, Steven H. Lin

**Affiliations:** 1 Department of Radiation Oncology, The University of Texas MD Anderson Cancer Center, Houston, Texas, USA; 2 Department of Radiotherapy, Erasmus Medical Center Cancer Institute, Rotterdam, the Netherlands; 3 Department of Radiation Oncology, Amsterdam UMC, Amsterdam, the Netherlands; 4 Department of Biostatistics and Data Science, University of Texas Health Science Center, Houston, Texas, USA; 5 Department of Population Health, The University of Austin Dell Medical School, Austin, Texas, USA; 6 Department of Radiation Physics, The University of Texas MD Anderson Cancer Center, Houston, Texas, USA; 7 Department of Gastrointestinal Medical Oncology, The University of Texas. MD Anderson Cancer Center, Houston, Texas, USA; 8 Department of Thoracic and Cardiovascular Surgery, The University of Texas MD Anderson Cancer Center, Houston, Texas, USA

**Keywords:** Esophageal cancer, 90-day mortality, Esophagectomy, Chemoradiation, Proton-beam therapy

## Abstract

**Purpose:**

Evidence suggests that proton-beam therapy (PBT) results in less toxicity and postoperative complications compared to photon-based radiotherapy in patients who receive chemoradiotherapy followed by esophagectomy for cancer. Ninety-day mortality (90DM) is an important measure of the postoperative (nononcologic) outcome as proxy of quality-of-care. We hypothesize that PBT could reduce 90DM compared to photon-based radiotherapy.

**Materials and Methods:**

From a single-center retrospective database patients treated with chemoradiotherapy before esophagectomy for cancer were selected (1998-2022). Univariable logistic regression was used to study the association of radiotherapy modality with 90DM. Three separate methods were applied to adjust for confounding bias, including multivariable logistic regression, propensity score matching, and inverse probability of treatment weighting. Stratified analysis for the age threshold that maximized the difference in 90DM (ie, ≥67 vs <67 years) was performed.

**Results:**

A total of 894 eligible patients were included and 90DM was 5/202 (2.5%) in the PBT versus 29/692 (4.2%) in the photon-based radiotherapy group (*P* = .262). After adjustment for age and tumor location, PBT versus photon-based radiotherapy was not significantly associated with 90DM (*P* = .491). The 90DM was not significantly different for PBT versus photon-based radiotherapy in the propensity score matching (*P* = .379) and inverse probability of treatment weighting cohort (*P* = .426). The stratified analysis revealed that in patients aged ≥67 years, PBT was associated with decreased 90DM (1.3% vs 8.8%; *P* = .026). Higher age significantly increased 90DM risk within the photon-based radiotherapy (8.8% vs 2.7%; *P* = .001), but not within the PBT group (1.3% vs 3.2%; *P* = .651).

**Conclusion:**

No statistically significant difference was observed in postoperative 90DM after esophagectomy for cancer between PBT and photon-based neoadjuvant chemoradiotherapy. However, among older patients a signal was observed that PBT may reduce 90DM risk.

## Introduction

Esophageal cancer is a common and frequently fatal type of cancer, with worldwide more than 500,000 estimated deaths per year.^1^ The standard of treatment for locally advanced esophageal cancer consists of neoadjuvant chemoradiotherapy followed by surgery in operable patients.^2^ This strategy results in a 10-year overall survival rate of 38%.

Esophagectomy carries a greater risk of postoperative mortality compared to other oncologic resections, with ≤5% as a suggested present-day quality benchmark.^3^ Historically, 30-day mortality was most frequently reported to evaluate the incidence of postoperative deaths.^3^, ^4^, ^5^, ^6^ However, the clinicians recognized that a considerable number of postoperative deaths occurred >30 days after the surgery.^5^, ^7^ Therefore, the 90-day mortality was suggested to provide a better definition of postoperative deaths and is nowadays one of the most important tools to measure the quality of the surgery.^3^, ^4^, ^5^, ^6^ The 90-day mortality has improved considerably over the years, from 7% to 9% in earlier trials,^5^, ^6^, ^8^ to 4% in more recent studies.^4^ Possible predictors of 90-day mortality reported in the literature include higher age, lower body mass index, cardiac comorbidity, squamous cell carcinoma histology, neoadjuvant chemoradiotherapy, lower hospital volume (ie, number of esophagectomies per year per center), positive surgical margins and an open surgical approach.^4^, ^8^

As mentioned, it is hypothesized that neoadjuvant chemoradiotherapy might increase the chance of postoperative mortality compared to no neoadjuvant therapy or chemotherapy only.^4^ However, most of this data is based on the patients treated with photon-based radiotherapy. Protons have better physical characteristics due to their steep dose gradient, lowering the integral body dose. As such, proton-beam therapy (PBT) can reduce the dose on vital structures like the heart and lungs, potentially lowering complication risk.^9^, ^10^ Recently, our group demonstrated in a randomized phase II trial of PBT versus photon-based radiotherapy that PBT reduces the total toxicity burden compared to photon therapy in patients with esophageal cancer.^11^ The difference in total toxicity burden was mostly driven by a difference in postoperative complications, with the most pronounced numeric differences in postoperative acute respiratory distress syndrome, atrial fibrillation, reintubation, and pneumonia. However, due to relative rarity of 90-day postoperative mortality, this trial with 107 patients was too small to detect potential subtle 90-day mortality differences between PBT and photon-based radiotherapy.

A retrospective multi-institutional analysis of radiation modality and postoperative outcomes in 580 esophageal cancer patients found no statistically significant difference in 90-day mortality between radiation modalities.^12^ According to this study the prevalence of 90DM was assumed to be 0.9% in the PBT group and 4.2% in the photon-based radiotherapy group, with a ratio of total patient number in PBT versus photon-based radiotherapy of 1:4. Assuming these numbers and an alpha of 0.05 and power of 0.80, to detect a statistically significant difference between both groups a sample size of 139 PBT and 556 photon-based radiotherapy patients would be required. In the current study, we aim to provide such a sufficient sample size.

Since PBT reduces the total toxicity burden in patients with esophageal cancer, our hypothesis is that PBT could reduce the incidence of 90-day postoperative mortality compared to photon-based radiotherapy. Therefore, the aim of this study was to compare the 90-day mortality after esophagectomy for esophageal cancer after proton-based versus photon-based neoadjuvant chemoradiotherapy.

## Materials and methods

This single-center retrospective cohort study was approved by our institutional review board (protocol number RCR02-542). The requirement to obtain informed consent was waived.

### Study population

From an institutional (single-center) database, patients with biopsy-proven esophageal cancer between 1998 and 2022 were selected. Inclusion criteria consisted of patients treated with neoadjuvant chemoradiotherapy followed by esophagectomy. Neoadjuvant radiotherapy generally consisted of a total dose of 50.4 Gy in 28 fractions delivered by either photon-based radiotherapy techniques (ie, three-dimensional conformal radiation therapy, intensity modulated radiation therapy or volumetric modulated arc therapy) or PBT techniques (ie, passive scattering proton therapy or intensity-modulated proton therapy). Concurrent chemotherapy generally consisted of a taxane-, platinum-, or fluoropyrimidine-based doublet regimen. Standard surgery consisted of an esophagectomy. Patients who did not complete chemoradiotherapy up to a dose of at least 41.4 Gy or with <90 days follow-up after surgery were excluded.

### Data collection and outcome

Information on baseline patient-, tumor- and treatment-related characteristics was extracted from the institutional database. The primary outcome consisted of all-cause mortality from day 0 up to (and including) day 90 after surgery.

### Statistical analysis

Baseline characteristics were compared between the PBT and photon-based radiotherapy groups. Nominal categorical variables were compared using the χ^2^ or Fisher’s exact tests and ordered variables using Mann-Whitney *U* tests. Parametric and nonparametric continuous variables were compared using Student’s *t* test or Mann-Whitney *U* test, respectively. Univariable logistic regression analyses were performed to study the association of radiotherapy modality as well as other patient-, tumor-, and treatment-related characteristics with 90-day postoperative mortality.

A serious risk of confounding by indication was expected as PBT was applied only in patients with insurance approval (eg, through health insurance plans in patients of any age or Medicare for patients aged ≥65 years). Three methods were applied to adjust for this confounding bias in separate analyses. First, after univariable logistic regression analyses, a multivariable logistic regression model was created in which the effect of PBT and photon-based radiotherapy on 90-day mortality was adjusted for other independent predictors. Significant variables from univariable analysis were entered in multivariable analysis. In case of collinearity, only the most significant variable was entered. Second, pretreatment imbalances between the 2 groups were corrected using 1:1 nearest-neighbor propensity score matching (PSM). Propensity scores were calculated based on all studied variables. A caliper of 0.25 of the standard deviation of the logit of the propensity score was used. The balance was judged well in case all standardized mean differences were <0.10. Third, inverse probability of treatment weighting (IPTW) was applied. The IPTW method is also based on propensity scores and calculates a weight for each subject that equals the inverse of the propensity score.^13^ These weights were applied to the study population to create a pseudopopulation in which confounders are more equally distributed.

In a final analysis, potential effect modification by patient age was studied through a stratified analysis for younger versus older patients. The threshold to split the group in these 2 age categories was determined by maximizing the difference in 90-day mortality between PBT versus photon-based radiotherapy. Chi-square and Fisher’s exact tests were used to compare 90-day mortality for protons versus photons within the different age groups. Also, the influence of patient age on 90-day mortality was determined within the PBT group and within the photon-based radiotherapy group separately. Statistical analyses were performed using R 3.6.3 open-source software (http://www.R-project.org; ‘rms’, ‘MatchIt’ package) and SPSS (version 28.0, IBM Corp., Armonk, New York). A *P*-value of <.05 was considered statistically significant.

## Results

**Figure 1:**
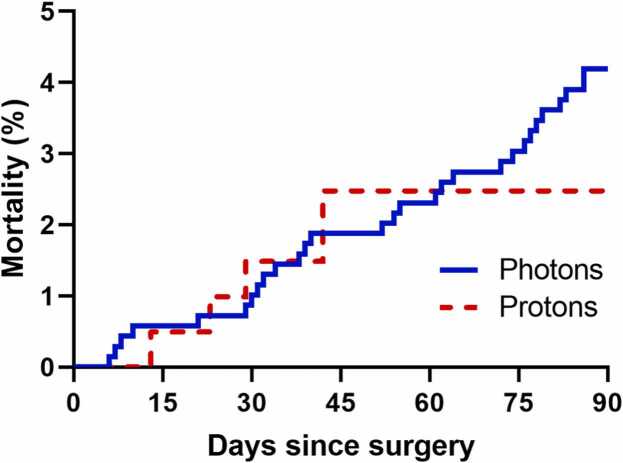
Cumulative postoperative mortality up to 90 days after previous PBT versus photon-based neoadjuvant chemoradiotherapy in patients with esophageal cancer.

A total of 894 eligible patients treated in our institution with esophageal cancer were included and divided in a PBT (*n* = 202) and photon-based radiotherapy (*n* = 692) group. In the proton group, 193 patients (96.6%) were treated with passive scattering proton therapy and 9 (4.5%) with intensity-modulated proton therapy. In the photon group, 313 patients (45.2%) were treated with intensity modulated radiation therapy, 224 (32.3%) with three-dimensional conformal radiation therapy, and 155 (22.4%) with volumetric modulated arc therapy. The baseline characteristics and a comparison of the proton and photon groups are presented in Table 1. Patients in the PBT group had a significantly higher age, better performance score, more hypertension, reflux and diabetes, a higher number of comorbidities, less induction chemotherapy, were generally treated in more recent years (especially more photon-based radiotherapy patients treated between 1998 and 2006 compared to PBT), and were treated more uniformly with 50.4 Gy in 28 fractions. The overall 90-day mortality rate was 5 (2.5%) in the PBT group and 29 (4.2%) in the photon-based radiotherapy group (Figure 1), which was not significantly different (*P* = .262). Stratification according to subgroups of patients who received induction chemotherapy versus those who did not, had clinical T1-2 versus T3-4 stage, or clinical N0 versus N1-3 disease, yielded no significant differences between PBT and photon-based radiotherapy. No significant difference in terms of cardiac or pulmonary complications between the PBT and photon-based therapy group was found.

**Table 1:** Baseline characteristics.

	*Before PSM*			*After PSM*			
Characteristic	Photon therapy (*n* = 692)	Proton therapy (*n* = 202)	*P* value	Photon therapy (*n* = 181)	Proton therapy (*n* = 181)	SMD	*P* value
Male sex	601 (86.8%)	178 (88.1%)	.636	165 (91.2%)	161 (89.0%)	0.074	.482
Age (years)	59.3 ± 9.8	62.6 ± 9.8	<.001*	61.6 ± 8.9	62.1 ± 10.0	0.054	.609
Performance status			.013*			<0.001	1.000
WHO 0	277 (40.0%)	98 (48.5%)		85 (47.0%)	85 (47.0%)		
WHO 1	391 (56.5%)	103 (51.0%)		96 (53.0%)	96 (53.0%)		
WHO 2	24 (3.5%)	1 (0.5%)		0 (0.0%)	0 (0.0%)		
BMI (kg/m^2^)	25.9 ± 5.3	26.7 ± 5.2	.064	26.8 ± 5.4	26.7 ± 5.1	0.011	.919
Hypertension	310 (44.8%)	121 (59.9%)	<.001*	108 (59.7%)	104 (57.5%)	0.045	.670
Cardiac comorbidity	107 (15.5%)	40 (19.8%)	.143	29 (16.0%)	32 (17.7%)	0.044	.674
Pulmonary comorbidity	44 ( 6.4%)	19 ( 9.4%)	.137	13 ( 7.2%)	15 ( 8.3%)	0.041	.694
Reflux	248 (35.8%)	94 (46.5%)	.006*	81 (44.8%)	82 (45.3%)	0.011	.916
Diabetes mellitus	96 (13.9%)	40 (19.8%)	.039*	33 (18.2%)	35 (19.3%)	0.028	.788
History of second malignancy	112 (16.2%)	42 (20.8%)	.127	33 (18.2%)	39 (21.5%)	0.083	.430
Tumor location			.090			0.054	.610
Upper/middle third	55 (7.9%)	9 ( 4.5%)		7 (3.9%)	9 (5.0%)		
Lower third	637 (92.1%)	193 (95.5%)		174 (96.1%)	172 (95.0%)		
Histology			.909			0.043	.684
Adenocarcinoma	627 (90.6%)	185 (91.6%)		169 (93.4%)	167 (92.3%)		
SCC	61 ( 8.8%)	16 (7.9%)		12 (6.6%)	14 (7.7%)		
Other	4 (0.6%)	1 (0.5%)		0 (0.0%)	0 ( 0.0%)		
Clinical T-stage			.965			0.059	.867
1-2	90 (13.0%)	25 (12.4%)		21 (11.6%)	23 (12.7%)		
3	586 (84.9%)	173 (86.1%)		158 (87.3%)	155 (85.6%)		
4	14 (2.1%)	3 (1.5%)		2 (1.1%)	3 (1.7%)		
Clinical N-stage			.681			0.068	.862
0	254 (37.0%)	67 (33.8%)		67 (37.0%)	63 (34.8%)		
1	267 (38.9%)	85 (42.9%)		71 (39.2%)	77 (42.5%)		
2-3	166 (24.1%)	46 (23.3%)		43 (23.8%)	41 (22.7%)		
Overall clinical stage			.841			0.042	.873
I	23 (3.4%)	8 (4.0%)		6 (3.3%)	7 (3.8%)		
II	251 (36.6%)	69 (34.8%)		69 (38.1%)	66 (36.5%)		
III	411 (60.0%)	121 (61.2%)		106 (58.6%)	108 (59.7%)		
Induction chemotherapy	289 (41.8%)	54 (26.7%)	<.001*	55 (30.4%)	50 (27.6%)	0.061	.563
Prescribed total dose (Gy)	50.4 [41.4-63.0]^a^	50.4 [50.4-50.4]^a^	<.001*	50.4 [50.4-50.4]^a^	50.4 [50.4-50.4]^a^	<0.001	1.000
Prescribed dose per fraction (Gy)	1.80 [1.80-2.25]^a^	1.8 [1.8-1.8]^a^	.060	1.8 [1.8-1.8]^a^	1.8 [1.8-1.8]^a^	<0.001	1.000
Prescribed number of fractions	28 [23-33]^a^	28 [28]^a^	<.001*	28 [28]^a^	28 [28]^a^	<0.001	1.000
Year of treatment			<.001*			0.047	0.906
1998-2006	267 (38.6%)	1 (0.5%)		0 (0.0%)	0 (0.0%)		
2007-2010	160 (23.1%)	50 (24.8%)		51 (28.2%)	50 (27.6%)		
2011-2014	118 (17.1%)	87 (43.0%)		70 (38.7%)	74 (40.9%)		
2015-2022	147 (21.2%)	64 (31.7%)		60 (33.1%)	57 (31.5%)		

**Abbreviations:**BMI, body mass index;PSM, propensity score matching; SCC, squamous cell carcinoma; SMD, standardized mean difference.

*Statistically significant (*P < .05*).

a Minimum-maximum range.

In univariable logistic regression analysis, PBT versus photon-based radiotherapy was not significantly associated with 90-day mortality (odds ratio [OR] 0.58, 95% confidence interval [CI] 0.22-1.52; Table 2). Significant univariable predictors of 90-day mortality included higher age (OR 1.08, 95% CI 1.03-1.13), tumor location in the upper/middle esophagus (OR 3.65, 95% CI 1.53-8.75), and nonadenocarcinoma histology (OR 2.71, 95% CI 1.14-6.44). Due to the collinearity between tumor location and histology, only tumor location was entered in multivariable analysis. After multivariable adjustment for age and tumor location, PBT versus photon-based radiotherapy was not associated with 90-day mortality (adjusted OR 0.49, 95% CI 0.18-1.31). Instead, only higher age (adjusted OR 1.08, 95% CI 1.03-1.13) and tumor location in the upper and middle third of the esophagus (adjusted OR 2.90, 95% CI 1.19-7.09) remained independently and significantly predictive of 90-day mortality.

**Table 2:** Univariable and multivariable logistic regression analysis for 90-day postoperative mortality.

	Univariable analysis		Multivariable analysis	
Characteristic	Crude OR (95% CI)	*P* value	Adjusted OR (95% CI)	*P* value
Male sex	2.42 (0.57-10.2)	.230		
Age (per 1 year)	1.08 (1.03-1.13)	.001*	1.08 (1.03-1.13)	<.001*
Performance status				
WHO 0	Reference			
WHO 1	1.99 (0.91-4.34)	.086		
WHO 2	3.54 (0.72-17.3)	.119		
BMI (per 1 kg/m^2^)	1.06 (0.99-1.13)	.054		
Hypertension	1.22 (0.61-2.42)	.574		
Cardiac comorbidity	1.88 (0.86-4.12)	.113		
Pulmonary comorbidity	2.38 (0.89-6.39)	.084		
Reflux	0.88 (0.43-1.79)	.717		
Diabetes mellitus	1.47 (0.63-3.45)	.376		
Number of comorbidities				
0	Reference			
1	2.20 (0.71-6.85)	.173		
≥2	2.44 (0.81-7.36)	.112		
History of second malignancy	1.03 (0.42-2.53)	.947		
Tumor location				
Lower third	Reference			
Upper/middle third	3.65 (1.53-8.75)	.004*	2.90 (1.19-7.09)	.020*
Histology				
Adenocarcinoma	Reference			
SCC or other	2.71 (1.14-6.44)	.024*		
Clinical T-stage				
1-2	Reference			
3-4	1.12 (0.39-3.23)	.840		
Clinical N-stage				
0	Reference			
1	1.48 (0.66-3.31)	.339		
2-3	1.22 (0.47-3.14)	.681		
Overall clinical stage				
I	Reference			
II	0.97 (0.12-7.82)	.975		
III	1.36 (0.18-10.4)	.770		
Induction chemotherapy	1.45 (0.73-2.89)	.290		
Prescribed total dose (Gy)	0.96 (0.83-1.10)	.541		
Prescribed dose per fraction (per 0.1 Gy)	1.35 (0.82-2.23)	.236		
Prescribed number of fractions	0.84 (0.65-1.08)	.179		
Year of treatment				
1998-2006	Reference			
2007-2010	0.58 (0.23-1.45)	.246		
2011-2014	0.42 (0.15-1.18)	.100		
2015-2022	0.58 (0.23-1.45)	.242		
Radiotherapy modality				
Photon-based radiotherapy	Reference			
PBT	0.58 (0.22-1.52)	.268	0.49 (0.18-1.31)	0.491

**Abbreviations:** BMI, body mass index; CI, confidence interval; OR, Odds ratio; PBT, proton-beam therapy; SCC, squamous cell carcinoma.

*Statistically significant (*P < .05*).

After PSM, a group of 181 PBT patients and 181 photon-based radiotherapy patients remained, and the groups were judged well balanced in baseline (Table 1). In the PSM groups the overall 90-day mortality was 5 (2.8%) in the PBT and 6 (3.3%) in the photon-based radiotherapy group, which was not significantly different (*P* = .379). After applying weights to patients in IPTW analysis, 2 outliers (ie, with very large weights) were excluded. After IPTW the groups were generally well balanced (Supplementary Table S1). The 90-day mortality rates in the IPTW cohort were 2.8% for proton and 4.1% for photon-based radiotherapy (*P* = .426), as shown in Supplementary Table S2.

All patients from the original cohort (*n* = 894) were stratified into a group with age <67 years and a group with age ≥67 years, as this was found as the ideal threshold (Supplementary Table S3). In the older group, PBT was associated with a decreased risk of 90-day postoperative mortality compared to photon-based radiotherapy (1.3% vs 8.8%, respectively, *P* = .026), but not in the younger group (2.8% vs 3.3%, respectively, *P* = .762), as shown in Table 3. Within the photon therapy group, age ≥67 years was related to a significantly increased risk of 90-day mortality compared to age <67 years (8.8% vs 2.7%, respectively, *P* = .001), whereas within the PBT group no such difference was observed (1.3% vs 3.2%, respectively, *P* = .651).

**Table 3:** 90-day mortality for different comparative groups of photon-based radiotherapy versus PBT.

Outcomes	Photon therapy	Within photon group *P* value	Proton therapy	Within proton group *P* value	Between group *P* value
*Before PSM*					
90-day mortality	29 / 692 (4.2%)	-	5 / 202 (2.5%)	-	.262
90-day mortality by age		.001*		.651	
<67 years	14 / 522 (2.7%)		4 / 125 (3.2%)		.762
≥67 years	15 / 170 (8.8%)		1 / 77 (1.3%)		.026*
*After PSM*					
90-day mortality	6 / 181 (3.3%)	-	5 / 181 (2.8%)	-	.379
90-day mortality by age		.062		.653	
<67 years	2 / 128 (1.6%)		4 / 114 (3.5%)		.331
≥67 years	4 / 53 (7.5%)		1 / 67 (1.5%)		.099

**Abbreviations:** 90d mort, 90-day mortality; PBT, proton-beam therapy; PSM, propensity score matching.

*Statistically significant (*P < .05*).

A comparable trend was observed in favor of PBT when a similar stratification for age was performed in the PSM cohort (90-day mortality 1.5% vs 7.5%, respectively, *P* = .099) and IPTW cohort (2.2% vs 9.1%, respectively, *P* = .124), but these differences were not statistically significant. In addition, within the photon therapy group of the PSM cohort (*n* = 181) a similar trend was observed that patients ≥67 years increased the risk of 90-day mortality compared to age <67 years (7.5% vs 1.6%, respectively, *P* = .062).

## Discussion

To date, the possible benefit of PBT over photon-based radiotherapy in terms of 90-day mortality is unknown. In our institutional experience, 90-day postoperative mortality after PBT was 2.5% versus 4.2% after photon-based radiotherapy. In logistic regression analysis as well as in PSM and IPTW comparisons, the 90-day postoperative mortality was not significantly different between PBT and photon-based radiotherapy. However, among older patients (≥67 years) we observed a signal that PBT may reduce the risk of 90-day mortality compared to photon-based radiotherapy from 8.8% to 1.3%. Also, higher age significantly increased the risk of 90-day mortality among patients who underwent photon-based radiotherapy, but not among patients who underwent PBT.

PBT has entered the modern-day treatment of esophageal cancer. The only randomized (phase II) trial completed to date showed in 107 esophageal cancer patients treated with PBT versus photon-based radiotherapy that the total toxicity burden was 2.3 times lower for PBT compared to photon therapy.^11^ Furthermore, patients treated with PBT had a 7.6 times lower risk of postoperative complications compared to patients treated with photons in the trial.^11^ The trial was underpowered to study a potential benefit in terms of 90-day postoperative mortality. In addition, in key large trials (ie, CROSS and Neo-Aegis trial) comparing neoadjuvant (photon-based) radiotherapy with no radiotherapy (ie, with no neoadjuvant treatment^14^ or perioperative chemotherapy^15^) surgical mortality was not higher in the radiotherapy groups. Therefore, the impact of radiotherapy remains uncertain, particularly when radiation modality is considered.

While awaiting the ongoing phase III randomized controlled trials (NRG GI-006 [NCT03801876] and PROTECT [NCT05055648]), the current study provides the best power and evidence into the potential benefit of PBT in terms of 90-day mortality.

Age is an important factor in decision-making and (surgical) risk assessment in patients with esophageal cancer. A meta-analysis that pooled data of 25 publications on 9531 patients after esophagectomy showed that age ≥70 years old (*n* = 2573) was associated with increased in patient mortality (pooled OR 1.87, 95% CI 1.54-2.26), as well as increased pulmonary complications (pooled OR 1.49, 95% CI 1.29-1.71) and cardiac complications (pooled OR 2.06, 95% CI 1.75-2.41).^16^ Different retrospective studies showed that the 90-day mortality was higher among patients ≥70^17^ or ≥75 years old^18^, ^19^ compared to younger patients. In the above-mentioned studies, most patients received neoadjuvant treatment and predominantly underwent photon-based radiotherapy. A study specifically looking at the outcomes after CRT in elderly esophageal cancer patients showed a statistically significant increased rate of severe radiation pneumonitis (grade ≥3) in the elderly group (≥80 years) versus younger patients (*P* = .003).^20^ Our study showed a signal that neoadjuvant treatment with PBT in elderly patients may result in a lower 90-day postoperative mortality. In fact, 90-day mortality after PBT appeared comparably low in both older and younger patients. This suggests that PBT might decrease the negative impact of neoadjuvant radiotherapy on surgical mortality risk and can be considered especially in elderly patients.

A few limitations apply to this study. Although one of the largest studies on this topic, the number of events (ie, 90-day mortality) is still low, especially after PSM. This increases the chance of a type II error (ie, a false-negative result). In addition, caution is needed interpreting the results of the subgroup analyses, as these were considerably underpowered. Another limitation is the retrospective and single-center nature of this study. As this was no prospective randomized intervention study, no inferences on causality between radiation modality and 90-day mortality can be made based on the current study. In addition, an important factor associated with 90-day mortality risk is hospital volume. Unfortunately, this could not be assessed in this study, because it was a single-center study with no low hospital volume comparison available. Generalizability of our results might be a concern because of the high volume single-center design of the study; for example, postoperative mortality may be higher in centers with less expertise in managing postoperative complications. Another important factor that in some reports appears associated with 90-day mortality risk is the surgical technique (ie, open vs minimally-invasive, transthoracic vs transhiatal). Regrettably, this information was not available in sufficient detail. Furthermore, it could be that there is a residual confounding indication due to the intrinsic reasons that some patients receive protons while others do not (eg, insurance differences according to age, social status, etc.). A comprehensive analysis of overall exposure to the health care system (billing codes) in the aftermath of PBT versus photon-based radiotherapy may be an interesting way to quantify how toxicities are different between the treatment groups.

## Conclusion

In conclusion, this study showed that the 90-day mortality after esophagectomy for cancer was not significantly different between neoadjuvant chemoradiotherapy using PBT versus photon-based radiotherapy. However, among older patients, a signal was observed that PBT may reduce the risk of 90-day postoperative mortality. Higher age increased the risk of 90-day mortality in patients who underwent photon-based radiotherapy, but not in patients who underwent proton-based therapy. This finding should be validated in ongoing and future randomized controlled trials and may be useful in patient selection for PBT.

## Author contributions

**Pim J.J. Damen**: Data curation, Formal analysis, Methodology, Writing - original draft; **Peter S.N. van Rossum**: Conceptualization, Formal analysis, Methodology, Supervision, Writing - original draft, Writing - review & editing; **Yiqing Chen**: Formal analysis, Methodology, Writing - review & editing; **Chike O. Abana**: Data curation, Writing - review & editing; **Zhongxing Liao**: Resources, Writing - review & editing; **Brian P. Hobbs**: Methodology, Supervision, Validation, Writing - review & editing; **Radhe Mohan**: Resources, Writing - review & editing; **Mariela Blum-Murphy**: Resources, Writing - review & editing; **Wayne L. Hofstetter**: Resources, Writing - review & editing; **Steven H. Lin**: Conceptualization, Methodology, Supervision, Writing - review & editing.

## Funding

No external funding was involved in this investigation.

## Declaration of Conflicts of Interest

The authors declare the following financial interests/personal relationships which may be considered as potential competing interests: Dr Steven H. Lin reports a relationship with Beyond Spring Pharmaceuticals that includes: board membership and funding grants. Dr Steven H. Lin reports a relationship with Nektar Therapeutics that includes: funding grants. Dr Steven H. Lin reports a relationship with STCube Pharmaceuticals that includes: board membership and funding grants. Dr Steven H. Lin reports a relationship with IntraOp Corporation that includes: funding grants. Dr Steven H. Lin reports a relationship with AstraZeneca that includes: board membership. Dr Steven H. Lin reports a relationship with XRAD Therapeutics that includes: consulting or advisory. If there are other authors, they declare that they have no known competing financial interests or personal relationships that could have appeared to influence the work reported in this paper.

## Data Availability Statement

All data generated and analyzed during this study are included in this published article (and its Supplementary Information files).

## Footnotes

Appendix A Supplementary data associated with this article can be found in the online version at doi:10.1016/j.ijpt.2024.100012.
