# Supplementary material for: Comparing 90-Day Postoperative Mortality After Neoadjuvant Proton-Based Versus Photon-Based Chemoradiotherapy for Esophageal Cancer
Source: Int J Part Ther. 2024 Apr 24;11:100012. doi: 10.1016/j.ijpt.2024.100012 (PMC11095098; doi:10.1016/j.ijpt.2024.100012)
Supplement: Supplementary file 1 — Supplementary material [file mmc1.docx]

| **Supplemental Table S1.** Baseline characteristics before and after inverse probability for treatment weighting (IPTW). | | | | | | | |
| --- | --- | --- | --- | --- | --- | --- | --- |
|  | *Before IPTW* | | |  | *After IPTW* | | |
| **Characteristic** | **Photon therapy (n=691)** | **Proton therapy (n=201)** | ***p* value** |  | **Photon therapy (n=691)** | **Proton therapy (n=128)** | ***p* value** |
| Male sex | 600 (86.8%) | 177 (88.1%) | 0.735 |  | 604 (87.3%) | 114 (89.0%) | 0.686 |
| Age (years)^†^ | 59.3 ± 9.8 | 62.6 ± 9.8 | **<0.001*** |  | 59.9 ± 9.6 | 59.8 ± 11.0 | 0.903 |
| Performance status^†^ | 25.9 ± 0.6 | 26.7 ± 5.2 | **0.007*** |  | 1.60 ± 0.6 | 1.55 ± 0.5 | 0.293 |
| BMI (kg/m^2^)^†^ | 25.9 ± 5.3 | 26.7 ± 5.2 | **0.048*** |  | 26.1 ± 5.4 | 27.0 ± 5.2 | 0.059 |
| Hypertension | 310 (44.8%) | 121 (59.9%) | **<0.001*** |  | 336 (48.5%) | 65 (50.4%) | 0.672 |
| Cardiac comorbidity | 106 (15.3%) | 40 (19.9%) | 0.153 |  | 111 (16.1%) | 19 (14.8%) | 0.646 |
| Pulmonary comorbidity | 44 ( 6.4%) | 19 ( 9.5%) | 0.178 |  | 48 ( 6.9%) | 10 ( 8.0%) | 0.637 |
| Reflux | 247 (35.7%) | 94 (46.8%) | **0.006*** |  | 262 (37.9%) | 59 (45.6%) | 0.077 |
| Diabetes mellitus | 96 (13.9%) | 40 (19.9%) | **0.048*** |  | 106 (15.3%) | 21 (16.3%) | 0.729 |
| Number of comorbidities  0  1  ≥2 | 169 (24.5%)  255 (36.9%)  267 (38.6%) | 39 (18.9%)  59 (29.4%)  104 (51.7%) | **0.004*** |  | 160 (23.2%)  244 (35.3%)  287 (41.5%) | 29 (22.5%)  43 (33.4%)  57 (44.2%) | 0.837 |
| Second malignancy | 111 (16.1%) | 42 (20.9%) | 0.135 |  | 118 (17.1%) | 23 (18.1%) | 0.750 |
| Tumor location  Upper/middle third  Lower third | 54 ( 7.8%)  637 (92.2%) | 9 ( 4.5%)  192 (95.5%) | 0.142 |  | 48 ( 7.0%)  643 (93.0%) | 6 ( 5.3%)  122 (94.7%) | 0.472 |
| Histology  Adenocarcinoma  SCC  Other | 627 (90.7%)  60 ( 8.7%)  4 ( 0.6%) | 184 (91.5%)  16 ( 8.0%)  1 ( 0.5%) | 0.939 |  | 629 (91.0%)  59 ( 8.5%)  4 ( 0.5%) | 118 (92.8%)  9 ( 6.9%)  1 ( 0.3%) | 0.631 |
| Clinical T stage  1-2  3  4 | 90 (13.1%)  585 (84.9%)  14 ( 2.0%) | 25 (12.5%)  172 (86.0%)  3 ( 1.5%) | 0.865 |  | 91 (13.1%)  586 (85.0%)  13 ( 1.9%) | 15 (12.1%)  111 (86.6%)  2 ( 1.3%) | 0.805 |
| Clinical N stage  0  1  2-3 | 254 (37.0%)  267 (38.9%)  165 (24.1%) | 67 (34.0%)  84 (42.6%)  46 (23.4%) | 0.623 |  | 248 (36.2%)  272 (39.7%)  165 (24.1%) | 46 (36.3%)  48 (38.2%)  32 (25.5%) | 0.922 |
| Overall clinical stage^†^ | 2.57 ± 0.6 | 2.57 ± 0.6 | 0.952 |  | 2.57 ± 0.6 | 2.57 ± 0.6 | 0.898 |
| Induction chemotherapy | 288 (41.7%) | 54 (26.9%) | **<0.001*** |  | 266 (38.5%) | 37 (29.0%) | 0.036 |
| Prescribed total dose (Gy)^†^ | 49.3 ± 2.7 | 50.4 ± 0.0 | **<0.001*** |  | 49.6 ± 2.4 | 50.4 ± 0.0 | **<0.001*** |
| Prescribed dose per fraction (Gy)^†^ | 1.8 ± 0.1 | 1.8 ± 0.0 | 0.075 |  | 1.8 ± 0.1 | 1.8 ± 0.0 | **0.001*** |
| Prescribed number of fractions^†^ | 27.3 ± 1.3 | 28 ± 0.0 | **<0.001*** |  | 27.5 ± 1.2 | 28.0 ± 0.0 | **<0.001*** |
| Year of treatment  1998-2006  2007-2010  2011-2014  2015-2022 | 267 (38.6%)  160 (23.2%)  118 (17.1%)  146 (21.1%) | 0 ( 0.0%)  50 (24.9%)  87 (43.3%)  64 (31.8%) | **<0.001*** |  | 207 (29.9%)  163 (23.6%)  161 (23.3%)  161 (23.2%) | 0 ( 0.0%)  46 (35.7%)  42 (32.6%)  41 (31.7%) | **<0.001*** |
| IPTW: Inverse probability of treatment weighting. PSM: propensity score matching. SCC: squamous cell carcinoma. SMD: standardized mean difference.  *: statistically significant (p<0.05)  ^†^: mean ± standard deviation | | | | | | | |

| **Supplemental Table S2.** 90-day mortality outcomes in inverse probability for treatment weighting (IPTW) cohort of photon-based radiotherapy vs. proton therapy. | | | |
| --- | --- | --- | --- |
| **Outcomes** | **Photon therapy** | **Proton therapy** | **Between group *p* value** |
| 90-day mortality | 29 / 691 (4.1%) | 4 / 128 (2.8%) | 0.426 |
| 90-day mortality by age  <67 years  ≥67 years | 12 / 513 (2.4%)  16 / 178 (9.1%) | 3 / 89 (3.0%)  1 / 39 (2.2%) | 0.701  0.124 |
|  | | | |

| **Supplemental Table S3.** Ninety-day mortality between photon and proton therapy groups stratified by age. | | | |
| --- | --- | --- | --- |
| **Age** | **Photon therapy**  **(n/n [%])** | **Proton therapy**  **(n/n [%])** | ***p* value** |
| 60 | 26/339 ( 7.1%) | 4/134 (2.9%) | 0.051 |
| 61 | 25/307 ( 7.5%) | 3/132 (2.2%) | **0.031*** |
| 62 | 20/278 ( 6.7%) | 3/129 (2.3%) | 0.065 |
| 63 | 19/257 ( 6.9%) | 3/118 (2.5%) | 0.096 |
| 64 | 18/226 ( 7.4%) | 3/113 (2.6%) | 0.091 |
| 65 | 18/202 ( 8.2%) | 3/101 (2.9%) | 0.090 |
| 66 | 17/170 ( 9.1%) | 2/ 86 (2.3%) | **0.042*** |
| 67 | 15/155 ( 8.8%) | 1/ 76 (1.3%) | **0.026*** |
| 68 | 17/546 ( 3.0%) | 4/138 (2.8%) | **0.041*** |
| 69 | 12/117 ( 9.3%) | 1/ 59 (1.7%) | 0.065 |
| 70 | 11/ 88 (11.1%) | 1/ 49 (2.0%) | 0.061 |
| *: statistically significant (*p*<0.05). | | | |
